# Biosynthesis and function of secondary metabolites

**DOI:** 10.3762/bjoc.7.190

**Published:** 2011-12-05

**Authors:** Jeroen S Dickschat

**Affiliations:** 1 Institut für Organische Chemie, Technische Universität Carolo-Wilhelmina zu Braunschweig, Hagenring 30, D-38106 Braunschweig, Germany

Natural products have long been used by humans owing to their beneficial effects. Indulgences such as coffee and tea, with their moderate stimulatory properties, have a long cultural tradition and are today some of the most important agricultural products worldwide. Other drugs have much stronger impacts on the central nervous system. For example, coca was originally used by the Inca civilisation because of its strong stimulatory effects, which result in feelings of happiness and euphoria. Opium was used by many ancient cultures as an efficient analgesic, but this also illustrates one of the first examples of drug abuse as it was used by warriors as an anxiolytic when girding their loins. Besides their pleasant traits these highly active drugs have adverse side effects, including most importantly an extremely high potential to cause addiction in combination with physical and mental prostration upon long-term abuse. Therefore, the production, importation, trading, and possession of these drugs are today under strict control by public authorities. Another historically interesting example of drug misuse is given by the death penalty imposed on Socrates in 399 B.C., carried out by the forced ingestion of the proverbial cup of hemlock.

These traditional and historical examples of the usage of bioactive natural products all originated in the ancient cultures, without any knowledge about the underlying chemistry. This situation started to change at the beginning of the 19^th^ century, when Friedrich Wilhelm Adam Sertürner (1783–1841) first isolated morphine from opium, naming it after Morpheus, the Greek god of dreams and demonstrating its activity in self-tests, as was typical practice during these early times of chemistry. A likewise important discovery was made by Alexander Fleming (1881–1955), who isolated the antibiotic penicillin from the mould *Penicillium notatum* in a groundbreaking contribution, which was awarded the Nobel Prize in 1945.

The discovery of new bioactive natural products is still a fascinating field in organic chemistry as demonstrated by the recent paradigms of the anticancer drug epothilon, the immunosuppressant rapamycin, or the proteasome inhibitor salinosporamide, to name but a few of hundreds of possible examples. Finding new secondary metabolites is a prerequisite for the development of novel pharmaceuticals, and this is an especially urgent task in the case of antibiotics due to the rapid spreading of bacterial resistances and the emergence of multiresistant pathogenic strains, which poses severe clinical problems in the treatment of infectious diseases. This Thematic Series on the biosynthesis and function of secondary metabolites deals with the discovery of new biologically active compounds from all kinds of sources, including plants, bacteria, and fungi, and also with their biogenesis. Biosynthetic aspects are closely related to functional investigations, because a deep understanding of metabolic pathways to natural products, not only on a chemical, but also on a genetic and enzymatic level, allows for the expression of whole biosynthetic gene clusters in heterologous hosts. This technique can make interesting, new secondary metabolites available from unculturable microorganisms, or may be used to optimise their availability by fermentation, for further research and also for production in the pharmaceutical industry. Especially fascinating is the intrinsic logic of the polyketide and nonribosomal peptide biosynthetic machineries, which is strongly correlated with the logic of fatty acid biosynthesis as part of the primary metabolism. Insights into the mechanisms of modular polyketide and nonribosomal peptide assembly lines open up the possibility for direct modifications, e.g., of oxidation states of the natural product’s carbon backbone by simple domain knockouts within the responsible megasynthases, or the introduction of a variety of alternative biosynthetic starters by mutasynthesis approaches, thus leading to new variants of known metabolites, which may have improved properties for therapeutic use. Another interesting aspect is the usage of enzymes in chemical transformations, which can provide synthetic organic chemists with an efficient access route to typically chiral building blocks that may otherwise be difficult to obtain.

It is my great pleasure to thank all of the contributors to this Thematic Series, the staff of the Beilstein-Institut, and my esteemed colleague and teacher Prof. Henning Hopf (Braunschweig) for opening the opportunity to guest-edit the present issue. Enjoy reading it!

Jeroen S. Dickschat

Braunschweig, October 2011

